# Medical Student Documentation in the Electronic Medical Record: Patterns of Use and Barriers

**DOI:** 10.5811/westjem.2016.10.31294

**Published:** 2016-12-15

**Authors:** Kathleen Wittels, Joshua Wallenstein, Rahul Patwari, Sundip Patel

**Affiliations:** * Harvard Medical School, Brigham and Women’s Hospital, Department of Emergency Medicine, Boston, Massachusetts; † Emory University School of Medicine, Department of Emergency Medicine, Atlanta, Georgia; ‡ Rush Medical College, Department of Emergency Medicine, Chicago, Illinois; § Cooper University Health Care, Department of Emergency Medicine, Camden, New Jersey

## Abstract

**Introduction:**

Electronic health records (EHR) have become ubiquitous in emergency departments. Medical students rotating on emergency medicine (EM) clerkships at these sites have constant exposure to EHRs as they learn essential skills. The Association of American Medical Colleges (AAMC), the Liaison Committee on Medical Education (LCME), and the Alliance for Clinical Education (ACE) have determined that documentation of the patient encounter in the medical record is an essential skill that all medical students must learn. However, little is known about the current practices or perceived barriers to student documentation in EHRs on EM clerkships.

**Methods:**

We performed a cross-sectional study of EM clerkship directors at United States medical schools between March and May 2016. A 13-question IRB-approved electronic survey on student documentation was sent to all EM clerkship directors. Only one response from each institution was permitted.

**Results:**

We received survey responses from 100 institutions, yielding a response rate of 86%. Currently, 63% of EM clerkships allow medical students to document a patient encounter in the EHR. The most common reasons cited for not permitting students to document a patient encounter were hospital or medical school rule forbidding student documentation (80%), concern for medical liability (60%), and inability of student notes to support medical billing (53%). Almost 95% of respondents provided feedback on student documentation with supervising faculty being the most common group to deliver feedback (92%), followed by residents (64%).

**Conclusion:**

Close to two-thirds of medical students are allowed to document in the EHR on EM clerkships. While this number is robust, many organizations such as the AAMC and ACE have issued statements and guidelines that would look to increase this number even further to ensure that students are prepared for residency as well as their future careers. Almost all EM clerkships provided feedback on student documentation indicating the importance for students to learn this skill.

## INTRODUCTION

Electronic health records (EHR) are commonly used in academic medical centers and provide advancement over traditional paper records in healthcare delivery. As EHRs have become more common, it is important to consider the implications of these systems on medical student education. The Liaison Committee on Medical Education (LCME) and the Association of American Medical Colleges (AAMC) have both identified communication as a key skill to be taught to medical students, including written communication.^1^,^2^ Further, AAMC has defined 13 Entrustable Professional Activities (EPA) that all medical students should attain by graduation. EPA 5 requires that the student be able to “document a clinical encounter in the medical record.”^3^

The Alliance for Clinical Education (ACE) published a statement in 2012 with the recommendations that students should have the opportunity to document in the EHR and that the notes should be reviewed.^4^ In this statement, they also recommended that students have the opportunity to practice entering orders in the EHR and that medical schools should have competencies related to charting in the EHR. In a previous survey of clerkship directors across specialties in 2009, 64% of students had access to their institutions EHR, and of those two-thirds had the ability to document. While EM clerkship directors were well represented in this multi-specialty study (26%), the study was somewhat limited by its low response rate of 32%.^5^

EHR use has grown significantly since 2009, in large part due to national incentives including those contained in the Affordable Care Act. While our published national EM curriculum recommends that students have the ability to document in the patient record,^6^ little is known about our current practices with regard to the EHR. Our objective was to better understand the frequency with which students are permitted to document in the EHR in EM clerkships and perceived barriers to student documentation. In addition, we examined current practices in the review of student notes and their use in feedback and assessment.

## METHODS

We conducted a cross-sectional study of EM clerkship directors at U.S. medical schools between March and May 2016. Eligible participants were members of Clerkship Directors in Emergency Medicine (CDEM). The Emory University Institutional Review Board reviewed our study protocol and determined that it was exempt from full committee review.

We developed a 13-question electronic survey that assessed student documentation during EM clerkships. The survey was designed for completion on an institutional level, and the survey instructions requested that only one survey be completed per clerkship. Participants were required to enter their name, title, and institution, and provide consent for use of responses for research purposes. Participants were asked if students in their clerkship document patient-care encounters at one or more clinical sites, and reasons why students do not document in the EHR were requested from those who indicated “no” to the preceding question. Six reasons for non-use that were expected by the authors to be common among clerkship directors were listed. Clerkship directors were asked to select one or more of the six that applied to their clerkship, or enter a free-text response.

For those who indicated that students do document in the EHR, the survey then addressed review of those notes for accuracy and/or student assessment, as well as the process of providing students feedback and assessment. The full survey can be accessed in our online appendix.

We sent out a link to the survey at the end of a lecture in the March 2016 Council of Residency Directors meeting where many EM clerkship directors were present. Subsequently, email invitations for the electronic survey (SurveyMonkey, Palo Alto, CA) were sent to current members of CDEM in March 2016, totaling 160 individuals representing 116 institutions. Second and third email reminders were sent to clerkship directors in early April 2016 using the CDEM as well as SAEM listservs to maximize response rate.

## RESULTS

Survey responses were received from 113 individuals. We excluded 13 entries as they were completed by a second clerkship (or assistant) director from within the same clerkship. This yielded 100 unique institutional entries, yielding a survey response rate of 86%. Three clerkships indicated that their sites do not use an EHR, and those surveys were excluded from further survey analysis. A representation of our survey distribution, response pattern, and exclusions can be seen in Figure 1.

Of the remaining 97 completed surveys, 61 clerkships (63%) indicated that students document patient care encounters in the EHR at one or more sites. Of the 36 clerkships (37%) that indicated their students do not document in the EHR, the most common reason cited was a hospital or medical school rule forbidding student documentation (80%). Table 1 details all reasons selected for not allowing medical student documentation in the EHR. There were four free-text responses that were closely aligned with our pre-selected choices (hospital policy - two, liability -one, not and educational objective - one) and did not reveal any additional reasons for non-use.

Almost all programs (95%) indicated that a portion of notes are reviewed for purposes of providing feedback. Details on the process of review, feedback, and evaluation can be found in Table 2.

## DISCUSSION

Nearly all clerkship directors surveyed indicated that EHRs are used in their departments, a significant increase from the 2009 ACE study. EM educators have recognized the importance of training in EHR documentation as almost two-thirds of clerkships allow students to document in the EHR. Nearly all programs that allow students to document in the EHR have a mechanism for review of notes, feedback, and assessment. There is significant variation in the patterns of review, feedback, and assessment among clerkships. This could be explained by variations in student/learner ratios between clerkships as well as other factors.

An examination of the barriers to student documentation in EHRs could provide an answer as to why the percentage of clerkships allowing EHR documentation is not even higher. Our study found that the most common reason students were not allowed to document in the EHR was due to hospital/medical school institutional policies. Given that 90% of medical school deans felt students should document in the chart and 93% felt that student education would be adversely affected if this were not allowed,^7^ there appears to be a disconnect between educational goals and institutional policies related to documentation. It is possible that some of these policies could be due to concerns over medical liability, which emerged as another major reason for non-use even if not explicitly prohibited by hospital policy. While difficult to reliably quantify, there does not seem to be significant evidence indicating a high liability risk specifically associated with medical student documentation. An extensive literature search using PubMed, Ovid Medline, and Google Scholar with the terms “medical student,” “documentation,” “malpractice” and “liability” did not reveal any studies or case reports on student documentation leading to malpractice. The one paper we found discussed the potential for a lawsuit due to student documentation but never cited a case.^8^

The additional cited barriers to use largely relate to intrinsic challenges faced by all EM clinicians and departments, particularly the need to balance education with clinical productivity, and the lack of available workspace in crowded departments. It is notable that of all clerkship directors who indicated non-use, only 15% related this to documentation not being an educational goal of the clerkship.

## LIMITATIONS

A number of limitations may affect our survey results and their interpretation. First, our CDEM-member eligible study participants represent a subset of EM clerkships and clerkship directors, whose policies and views may not be representative of all clerkships and leaders. This database was selected as our study population as the authors were unable to locate another database of EM clerkships in the U.S. that was felt to be accurate and/or current. The Liaison Committee of Medical Education maintains a list of accredited U.S. medical schools; however, not all of those schools have an academic EM department or clerkship. There are multiple databases of EM residency programs, though there are residency programs without affiliated clerkship programs, and clerkships without affiliated residency programs. While we have no reason to believe that clerkship programs whose leaders are CDEM members are not representative of all EM clerkships, this remains a confounding variable. Doctor of osteopathy programs comprise a small minority of the CDEM membership, so our findings may not represent practices at these programs. Second, as with all survey-based research, our respondents may have different characteristics and viewpoints than non-respondents.

## CONCLUSION

The large number of EM clerkships that allow students to document in the EHR and provide feedback on EHR use is well aligned with educational recommendations from within and beyond our specialty. An over-exaggerated fear related to medical liability may be a factor in preventing more widespread use. While there are certainly valid and legitimate barriers preventing more widespread use, we should search for solutions within our departments and advocate at an institutional level.

## Figures and Tables

**Figure:**
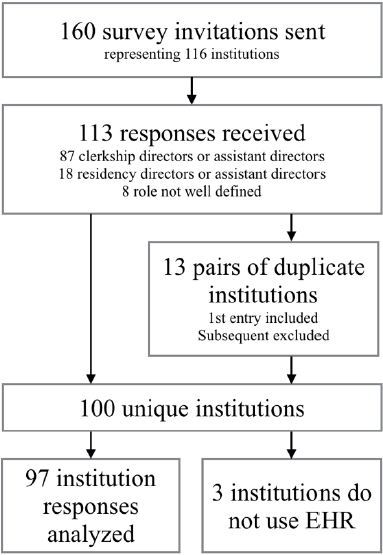
Representation of participants in a survey of medical student use of (EHR) electronic health records.

**Table 1:** Reasons cited for not allowing students to document in the EHR.

80%	Medical school or hospital rule forbidding student documentation
60%	Concern for medical liability
53%	Inability for student notes to support medical billing
38%	Lack of computer workspace/access
15%	No documentation educational objective for the clerkship
11%	Lack of ability to review notes and provide feedback

*EHR,* electronic health record

**Table 2:** Types of feedback provided on student documentation in electronic health record (EHR).

95% clerkships reviewed student notes for feedback
Fewer than half of notes reviewed (70%)
Half to three-quarters of notes reviewed (23%)
Three quarters or more reviewed (7%)
Feedback provided by faculty (92%)
Feedback provided by clerkship director (40%)
Feedback provided by residents (64%)
Oral feedback only provided (75%)
Oral and written feedback given (25%)
Documentation considered in final grade (58%)
